# UV Light Illumination Can Improve the Sensing Properties of LaFeO_3_ to Acetone Vapor

**DOI:** 10.3390/s18071990

**Published:** 2018-06-21

**Authors:** Heng Zhang, Hongwei Qin, Chengyong Gao, Guangjun Zhou, Yanping Chen, Jifan Hu

**Affiliations:** State Key Laboratory for Crystal Materials, School of Physics, Shandong University, Jinan 250100, China; 201411433@mail.sdu.edu.cn (H.Z.); gchy@sdu.edu.cn (C.G.); gjzhou@sdu.edu.cn (G.Z.); yanping_c@126.com (Y.C.)

**Keywords:** gas sensor, acetone vapor, UV light illumination, sol-gel method

## Abstract

The synthesized LaFeO_3_ nanocrystalline sensor powders show positive response to sensing acetone vapor at 200 °C. The responses to acetone vapor (at 0.5, 1, 2, 5, 10 ppm) are 1.18, 1.22, 1.89, 3.2 and 7.83. To make the sensor operate at a lower optimum temperature, UV light illumination 365 nm is performed. Response of the sensor has a larger improvement under 365 nm UV light illumination than without it. The responses to acetone vapor (at 0.5, 1, 2, 5, 10 ppm) are 1.37, 1.85, 3.16, 8.32 and 14.1. Furthermore, the optimum operating temperature is reduced to 170 °C. As the relative humidity increases, the resistance and sensitivity of sensor are reduced. The sensor shows good selectivity toward acetone when compared with other gases. Since the detection of ultralow concentrations of acetone vapor is possible, the sensor can be used to preliminarily judge diabetes in the general public, as a high concentration of acetone is exhaled in breath of diabetic patients. The sensor shows a good stability, which is further enhanced under UV light illumination. The sensor shows better stability when under 365 nm UV light illumination. Whether under light illumination or not. The LaFeO_3_ material shows good performance as a sensor when exposed to acetone vapor.

## 1. Introduction

Acetone is a widely used material in several industrial applications [1]. It is a colorless and transparent liquid with a mild fruity odor. Because of its low cost and versatility, acetone has a great application value in chemical industry. However, acetone is volatile and flammable, and can explode when exposed to fire. Acetone is also physiologically hazardous to humans and animals. For example, it can cause headache and nausea, and can damage the central nervous system upon long-term exposure [2]. Furthermore, acetone at ultralow concentration levels exist in exhaled breath. The concentration of acetone in exhaled breath from healthy and diabetic people are different [3], which, therefore, can be used as a marker to judge if a person is diabetic or not. Therefore, development of a sensor that can detect low or ultralow concentration of acetone is necessary. There are some detectors such as quartz crystal microbalances and fiber-optic sensors already existing in hospitals or special and health inspection agencies. However, the above-mentioned analytical tools are either inconvenient or expensive. There is a need for an acetone sensor with high sensitivity and stability that is also economical, such that detection of the concentration of acetone in exhaled breath or in air is possible.

In the past decades, metal semiconductor oxides have attracted much attention for their high sensitivity and stability towards detecting acetone. The list includes TiO_2_ [4,5,6], NiO [7,8], ZnO [9,10,11,12,13], Co_3_O_4_ [14], Fe_2_O_3_ [15,16], WO_3_ [17,18], and SnO_2_ [19,20,21,22,23], which have been reported as acetone sensors with good performance. In recent years, perovskite structures (ABO_3_) such as NdFeO_3_ [24], SmFe_1−x_Mg_x_O_3_ [25], Yb_1−x_Ca_x_FeO_3_ [26], LaNi_1−x_Ti_x_O_3_ [27], SmFeO_3_ [28], LaFeO_3_ [29,30] and La_0.75_Ba_0.25_FeO_3_ [31] have also shown good sensitivity, stability and selectivity toward acetone. The sensors above are list in Table 1.

Although there have been some reports on the use of the acetone sensing property of LaFeO_3_, no studies exist on the influence of ultraviolet light on the sensor. In this work, acetone vapor sensing performance of LaFeO_3_ is researched. The LaFeO_3_ displays maximum sensitivity at acetone concentrations of 1.18, 1.22, 1.89, 3.2 and 7.83 to 0.5, 1, 2, 5 and 10 ppm at 200 °C of optimum temperature, which is decreased by the use of 365 nm UV light illumination as sensor irradiation during the testing process. Our results show that UV irradiation not only decreases the optimum operating temperature, but also improves the sensitivity to acetone vapor. The responses are 1.37, 1.85, 3.16, 8.32 and 14.1 to the same concentration of acetone vapor at 170 °C when the sensor is irradiated by UV light. The dynamic resistance curve of the sensor material was studied and the mechanism of sensitivity improvement upon UV light illumination explained. Since good selectivity and extremely low detection limit are obtained, this sensor can be used to preliminarily judge if a person is diabetic or not by detecting the concentration of acetone in the exhaled breath. The stability of the sensor was also measured every 3 days for one month. The LaFeO_3_ still showed good response and stability among all the materials that were investigated under light illumination.

## 2. Materials and Methods

### 2.1. Preparation

The nanocrystalline LaFeO_3_ powders are synthesized by a sol-gel method. At the beginning, according to a certain chemical proportion of lanthanum nitrate, ferric nitrate, PEG (molecular weight 20,000) and nitric acid (all of analytical grade purity) are weighed and mixed in deionized water. An appropriate amount of nitric acid is necessary. Then the raw materials are mixed together in deionized water with the PH regulated at about 1.5–2. The mixture is heated in a water bath at 80 °C for two days with continuous stirring to get a highly viscous sol which becomes a gel in the next few hours. The gel is dried in an oven at 100 °C for 24 h to produce dried powder which is then grinded to form a fine powder. The powder is preheated at 400 °C for 2 h, following which it was annealed in at 800 °C for 4 h in a furnace. The LaFeO_3_ product is obtained by grinding.

### 2.2. Vapor Sensing Measurements

**Figure 1:**
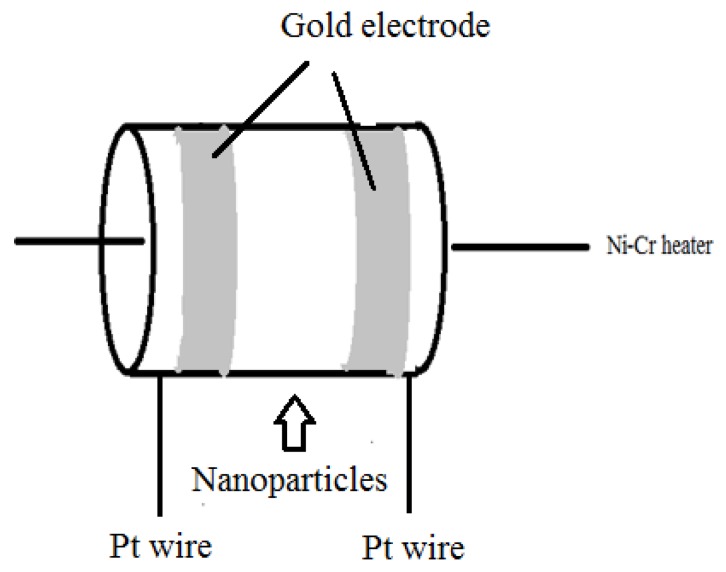
Schematic structure of the gas sensor.

LaFeO_3_ is blended with an appropriate amount of deionized water to shape a slurry. The sensor shown in Figure 1 is fabricated by coating the pastes onto a ceramic tube. The ready-made sensors are heated at 240 °C for 24 h on the aging equipment. The micro-injector is used to regulate the acetone vapor concentrations by injecting an appropriate amount of liquid acetone at first. The measuring instrument (WS-30A, Wei Sheng Electronic Technology Co., Ltd., Zhengzhou, China) is used to detect sensitivity of sensors. The complete preparation process is maintained at room temperature, and the room humidity is 20% RH. The vapor sensitivity performance *S* is defined as Rg/Ra. Ra is the resistance when the sensor is in air and the Rg is the resistance in the tested vapor. The response and recovery time are defined as the time taken to get 90% of the varied response after introducing and removing the tested vapor.

### 2.3. Characterization

X-ray diffractometer (XRD) using CuKα radiation is employed as a characterization technology to help us analyze the structure of the power. The element distribution is analyzed by energy-dispersive X-ray spectroscopy (EDAX). The nanostructure of LaFeO_3_ is analyzed by field emission scanning electron microscopy (FE-SEM).

## 3. Result and Discussion

**Figure 2:**
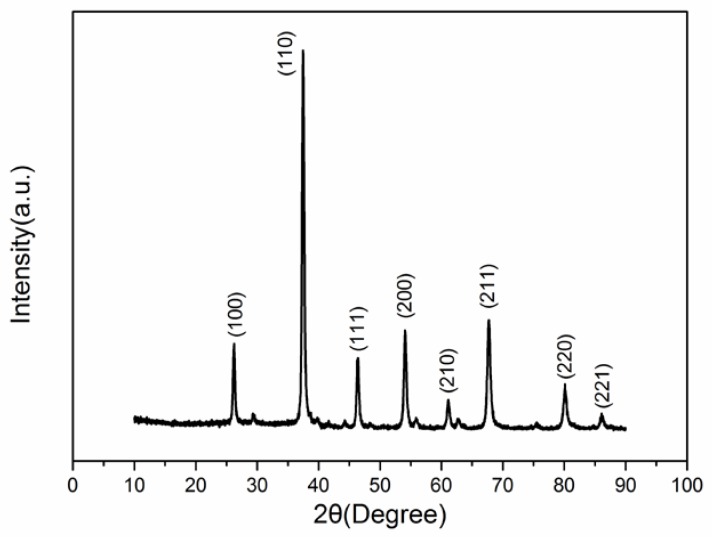
The XRD patterns of LaFeO_3_ (x = 0, 0.1, 0.2, 0.3) annealed at 800 °C.

**Figure 3:**
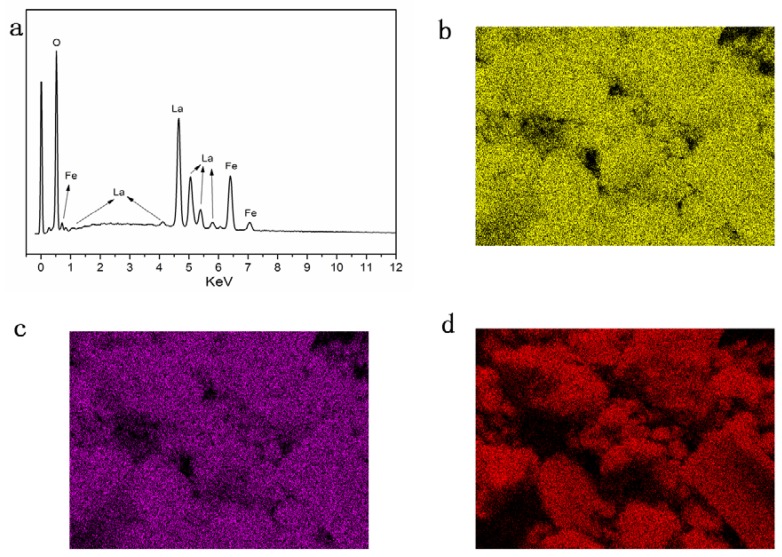
(**a**) EDS of LaFeO_3_; (**b**) La Mapping; (**c**) Fe Mapping; (**d**) O Mapping.

**Figure 4:**
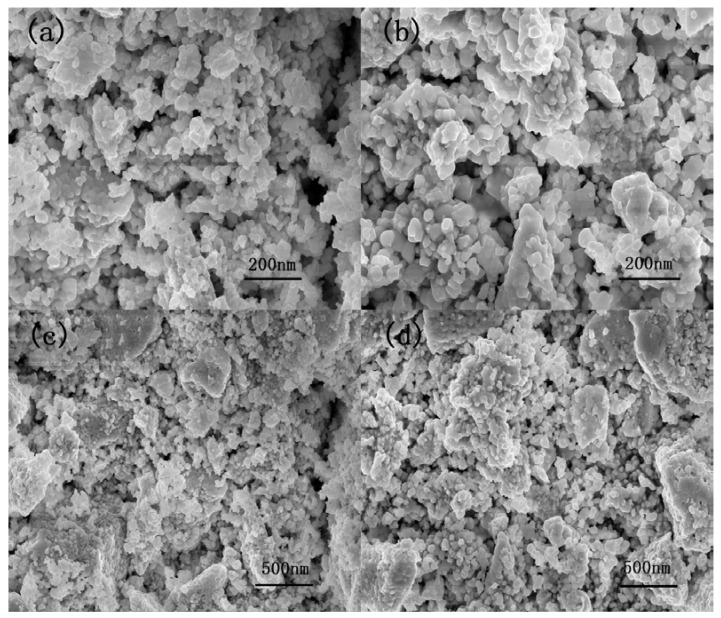
SEM images of LaFeO_3_ annealed at 800 °C. (**a**,**b**) The given scale is 200 nm; (**c**,**d**) The given scale is 500 nm.

Figure 2 shows the XRD of LaFeO_3_ material annealed at 800 °C. It is clear that the material shows a single perovskite structure in agreement with the standard PDF card: 75-0541. To make sure that there are no other elements in the semiconductor, EDS and EDAX mapping are performed, and the results are shown in Figure 3. It can be seen that the three elements above are contained in the material, and there are no other elements except O, Fe and La elements. Figure 4 shows the microstructure observed under different magnifications for LaFeO_3_ material annealed at 800 °C. The average particle size of the LaFeO_3_ material was estimated to be less than 80 nm.

**Figure 5:**
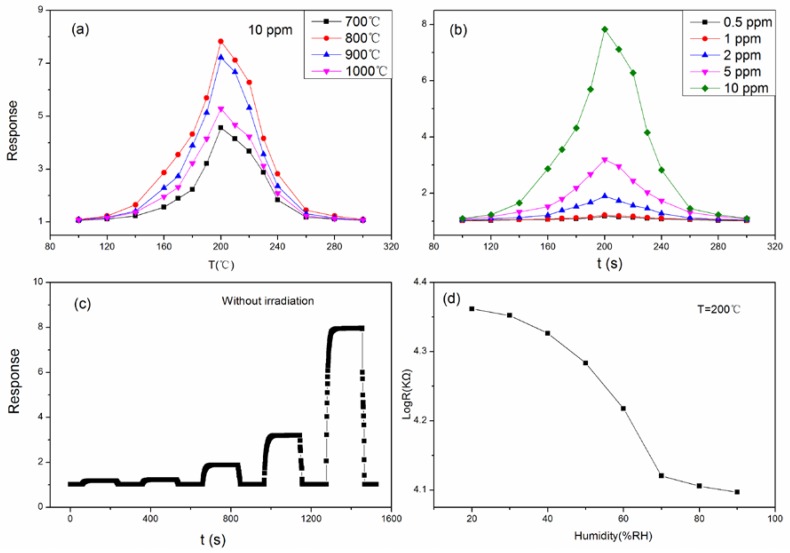
(**a**) The response of LaFeO_3_ with different annealing temperatures toward 10 ppm acetone vapor; (**b**) the sensitivity for LaFeO_3_ (with T_a_ = 800 °C) at different operating temperatures toward acetone vapor; (**c**) the dynamic curves of vapor sensitivity for LaFeO_3_ toward acetone vapor; (**d**) relationship between relative humidity and sensing resistance.

Figure 5a shows the sensing response of LaFeO_3_ annealed at different temperatures (700, 800, 900, 1000 °C) to 10 ppm acetone vapor. The responses are 4.56, 7.83, 7.22 and 5.28, respectively, at the optimum operating temperature of 200 °C. LaFeO_3_ annealed at 800 °C showed the maximum response. Next, we focused on investigating in detail the sensing proprieties of LaFeO_3_ material annealed at 800 °C. The responses obtained toward different concentrations of acetone vapor at different temperature are shown in Figure 5b. The maximum responses toward 0.5, 1, 2, 5 and 10 ppm acetone vapor are 1.18, 1.22, 1.89, 3.2 and 7.83, respectively. The optimum operating temperature for all measurements was 200 °C. The dynamic response curves for LaFeO_3_ to different concentration of acetone vapor at 200 °C are shown in Figure 5c. Since the response is define as Rg/Ra, the sensor is p-type if the response is greater than 1. The dynamic curves of the response are the same as the dynamic curves of the resistance of the sensor. The resistance of the sensor is kept relatively steady before acetone vapor is introduced. Upon the introduction of acetone vapor, the resistance increases rapidly. When the resistance reaches the maximum value, it soon maintains a relatively stable state. When the tested vapor is removed, the Rg/Ra response of the sensor decreases rapidly, which means the resistance of sensor decreases rapidly. All the sensors have a short response and recovery time. The response and recovery times of LaFeO_3_ to 10 ppm acetone vapor are 21 s and 6 s, respectively. There is adsorption oxygen on the sensor’s surface, adsorbed from the outside air. Meanwhile, the absorbed oxygen also desorbs from the sensor’s surface to the outside medium. However, the rate of oxygen absorption is greater than that for oxygen desorption at low temperature; therefore, much adsorption oxygen remains on the sensor’s surface. Adsorbed oxygen causes a series of chemical reactions to become anionic oxygen as shown through following chemical reactions:

O_2_ (gas) + e^−^ → O_2_^−^ (adsorption), (1)

O_2_^−^ (adsorption) + e^−^ → 2O^−^ (adsorption), (2)

2O^−^ (adsorption) + h^+^ → O_2_^−^ (adsorption), (3)

The h^+^ means the holes with positive charge.

After the acetone vapor is introduced, the oxygen species containing O^−^ and O_2_^−^ will react with acetone molecules, resulting in the increase of sensor sensitivity. With the rise in temperature, the rates of oxygen adsorption and desorption are improved, with the rate of adsorption becoming greater than that of desorption. Therefore, the amount of oxygen species increases on sensor’s surface. At 200 °C, the amount of oxygen species reaches the maximum value, which means that the chemical reaction between acetone molecules and oxygen species is the most efficient and the sensor shows maximum sensitivity toward acetone vapors. On the other hand, the energy from the rise in temperature provides the required activation energy for the reaction with the electron transfer in the chemical reaction to reach a maximum, which is also an important factor in improving the sensitivity toward acetone vapor. With a further increase in the temperature, the rate of oxygen desorption is improved by the heat energy from the rise in temperature. Additionally, the acetone vapor also begins to desorb from the surface of sensor. A small amount of acetone molecules also reacts with oxygen species, resulting in a declining trend in the sensor sensitivity. The following are the possible reactions occurring:

CH_3_COCH_3_ (vapor) + 8O^−^ (adsorption) → 3CO_2_ + 3H_2_O + 8e^−^, (4)

e^−^ + h^+^ → null + energy, (5)

The h^+^ means the holes with positive charge.

The dynamic resistance change curve of LaFeO_3_ in varied relative humidity is shown in Figure 5d. As the humidity increases, the resistance decreases. Especially from 40% to 70%, the decreasing trend is clear. Water molecules adsorbed on the surface of the sensor increase with the rise in humidity. At high temperature, the OH groups rather than H_2_O molecule exist on the surface of the semiconductor, and the other neutral H atom reacts with the lattice oxygen to form another OH and to form holes in the sensor. Concentration of holes increases with the increase in OH, decreasing the resistance of sensor.

**Figure 6:**
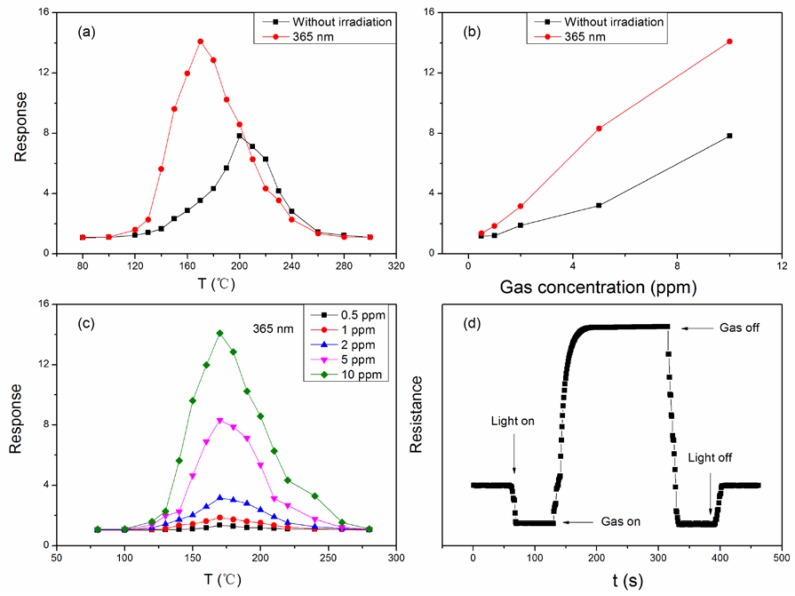
(**a**) Sensitivity of LaFeO_3_ to 10 ppm acetone vapor measured. (red): irradiated by 365 nm light; (black): without irradiation. (**b**) Response dependence on the acetone concentration at its optimum operating temperature. (**c**) The sensitivity for LaFeO_3_ (with T_a_ = 800 °C) measured toward acetone vapor under light illumination. (**d**) Dynamic resistance of LaFeO_3_ to acetone vapor at 170 °C under light.

The optimum operating temperature of 200 °C for the sensor is rather high, and then the acetone becomes dangerous. Therefore, from a practicality perspective, the optimum operation temperature of the sensor should be reduced. A UV-LED with 365 nm wavelength was introduced to irradiate the vapor sensor in this measurement system. The power consumption of the UV-LED was 50 mW. From Figure 6a, we can see that when the UV light is introduced, the optimum operating temperature is lower than that that without the UV light. The optimum operating temperature is 170 °C when the wavelength of the UV radiation is 365 nm. The smaller the wavelength, the lower the optimum operating temperature will be. In addition, compared with the natural environment, there is a big change in the response of the sensor when it is illuminated under UV light. The response of the sensor is 14.1 to 10 ppm acetone vapor when the wavelength is 365 nm, compared with a response of 7.83 in the natural environment. Light illumination has a positive effect and promotes the sensitivity of the sensor toward acetone. Figure 6b shows the response of the sensor with light illumination (red) and without irradiation (black) to different concentrations of acetone vapor at the optimum operating temperature. The response increases when the concentration of acetone increases. At lower concentrations of acetone vapor, the response of the sensor under UV light shows little difference compared to the response of the sensor without UV light illumination. However, the response of the sensor under UV light illumination increases more pronouncedly than that of the sensor without irradiation. Figure 6c shows the responses of the sensor under light illumination toward acetone vapor at different concentrations. The sensitivity is improved under the UV light illumination. Toward 0.5–10 ppm acetone, responses are 1.37, 1.85, 3.16, 8.32 and 14.1 under UV light illumination with a wavelength of 365 nm.

The dynamic curve of resistance for LaFeO_3_ under UV light illumination of 365 nm wavelength at 170 °C to acetone vapor of varying concentration is shown in Figure 6d. The resistance of the sensor was kept constant before the UV light was turned on. Upon the introduction of the UV light in the measuring system, the resistance soon starts to decrease rapidly and reach a stable value. The sensor absorbs energy from the light illumination and generates electron-hole pairs. The light-induced holes arrive on the surface of sensor, and capture electrons from the oxygen species, which will become oxygen molecules and desorb from the sensor surface. However, the number of oxygen species on the surface of the sensor is large; especially for p-type Fe-based perovskites [35], a lot of oxygen species will leave the sensor surface. When the acetone vapor is injected into the glass, the resistance will rise quickly and reach a stable value. The products thus produced by a chemical reaction between acetone molecules and the oxygen species will react with acetone molecules to trap electrons. The light-induced hole can trap OH^−^ from acetone to form OH•, which has strong oxidation capability. Thus, OH• can speed up the reaction between acetone molecules and oxygen species. As the number of holes decreases, resistance of the sensor increases. The plausible reactions are:

LaFeO_3_ + *hv* → e^−^ + h^+^, (6)

h^+^ + OH^−^ → OH•, (7)

h^+^ + O_2_^−^ → O•, (8)

CH_3_COCH_3_ + O_2_^−^ + OH• → CO_2_ + H_2_O + e^−^, (9)

**Figure 7:**
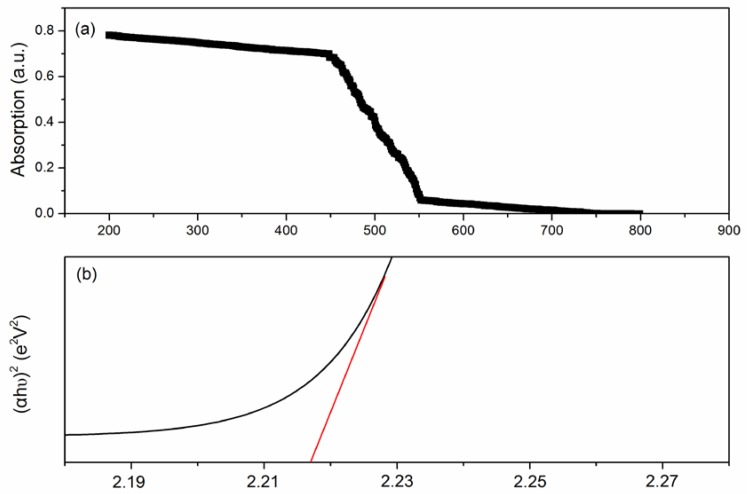
The UV−visible diffuse reflectance spectra (**a**), and the energy band gap (**b**) of the as-prepared LaFeO_3_ samples.

**Figure 8:**
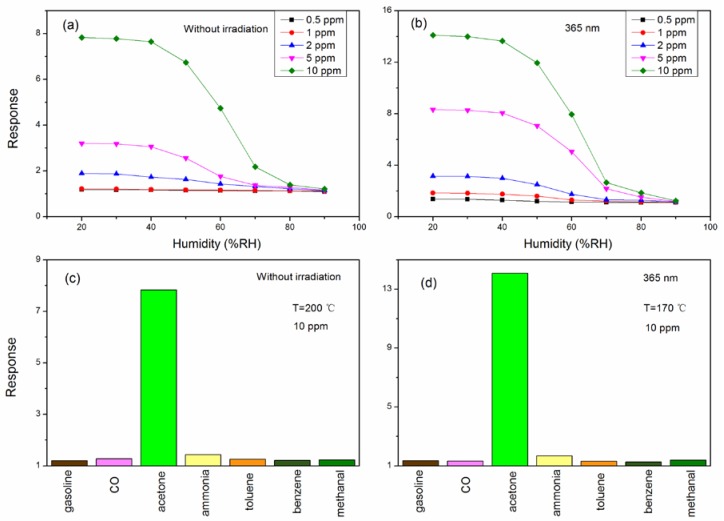
The sensitivity of the sensor toward acetone at different relative humidities: (**a**) Without irradiation at 200 °C; (**b**) 365 nm at 170 °C. The response of sensors based on LaFeO_3_ toward different 10 ppm vapors: (**c**) Without irradiation at 200 °C; (**d**) 365 nm at 170 °C.

Figure 7a shows the UV-visible diffuse reflectance spectra of LaFeO_3_. By calculation, Figure 8a is obtained. We can see that the band gap of LaFeO_3_ is about 2.218. The photon energy that the sensor can absorb is obtained through the formula: Eg = 1240/λ (λ is the wavelength of light). The photon energy is 3.39 eV (365 nm). The photon energy of the violet light (wavelength λ = 365 nm) is larger than the band gap of LaFeO_3_. Therefore, we can draw the conclusion that the sensitivity can be influenced or improved when the photons energy is larger than band gap of sensor.

Figure 8a,b shows the relationship between the humidity and the response of LaFeO_3_ to different concentrations of acetone vapor at its optimum operating temperature. We can see that the trend of response decreases whether the sensor is under light illumination or not. Especially at relative humidity of 50% to 70%, the decreasing trend is more pronounced. However, the responses of the irradiated sensor are greater than the responses of the sensor which is not exposed to irradiation at the same relative humidity when the relative humidity is below 80%. When the relative humidity is greater than 80%, there is almost no response to acetone vapor. The acetone sensing process is that O^−^ (adsorbed O species) reacts with acetone vapor, and the reaction products are CO_2_ and H_2_O. Therefore, with an increase in humidity, the reaction is slowed down and the response is reduced.

In the practical application process, many different kinds of gases may co-exist. As such, for accurate detection of acetone, the performance of the sensor should not be impacted by the presence of other gases. Therefore, evaluation of the performance and selectivity of the sensor in real-life situations is important. Figure 8c,d shows the selectivity of the sensor for acetone in the presence of different gases at 10 ppm. It can be seen form this figure that the sensor shows great selectivity toward acetone compared with other gases. The nano-particles of LaFeO_3_ adsorb acetone molecules with a large dipole moment (2.88 D). Additionally, the functional group in the acetone structure is broken rather easily. These factors both result in a high selectivity for the sensor toward acetone vapor.

**Figure 9:**
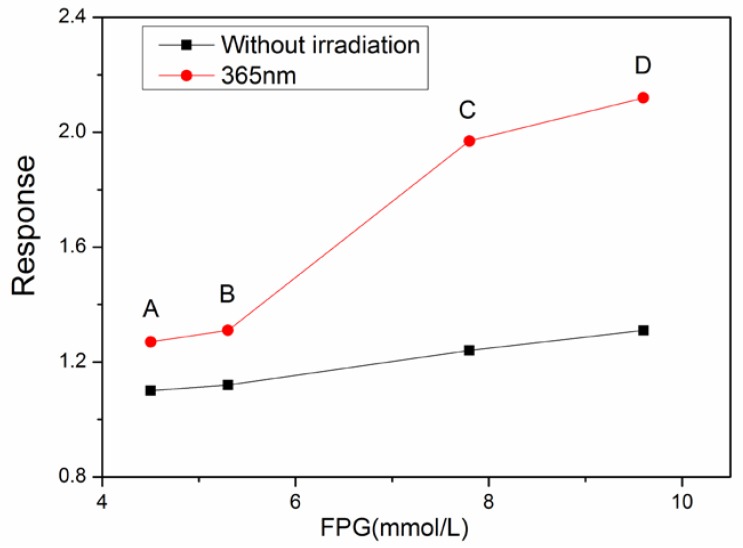
The response dependence on the concentration of FPG in four volunteers. A/B/C/D means the four volunteers.

To evaluate the real-life application of the sensor in detecting diabetes, the following experiment is performed. The concentration of acetone in exhaled breath from a healthy patient is about 0.3–0.9 ppm, and it can reach 1.8 ppm in people with diabetes. Four volunteers were invited to participate in real-life testing of the sensor. Their details are shown in Table 2. The first two are healthy, and are used as the references, and the other two are individuals with diabetes. FPG means concentration of fasting glucose. The four samples of exhaled breath from volunteers were kept in vacuum bags. During the measurement, the exhaled breath was injected within 5 s. As is known, there are 40,000 ppm of CO_2_ in exhaled breath, which should be subtracted from the final data. Other gases, like H_2_S, toluene, etc., are there in extremely low concentrations in exhaled breath. Therefore, the other gases above are ignored. Figure 9 shows the detection results of acetone by the sensor. All the data are as obtained after the sensitivity toward CO_2_ was subtracted. The comparison of acetone vapor sensitivity data in volunteers show that the acetone level is about 0.3 ppm in volunteer A, and 0.35 ppm in volunteer B, and for the last two volunteers, there is more than 1 ppm acetone in their exhaled breath. The results correspond very well with their basic health or diabetic condition. These results indicate that LaFeO_3_ is a potential sensor to preliminarily examine the concentration of acetone in exhaled breath. People who are detected as diabetic may then proceed to a hospital for a further detection and evaluation of concentration of acetone in their exhaled breath.

**Figure 10:**
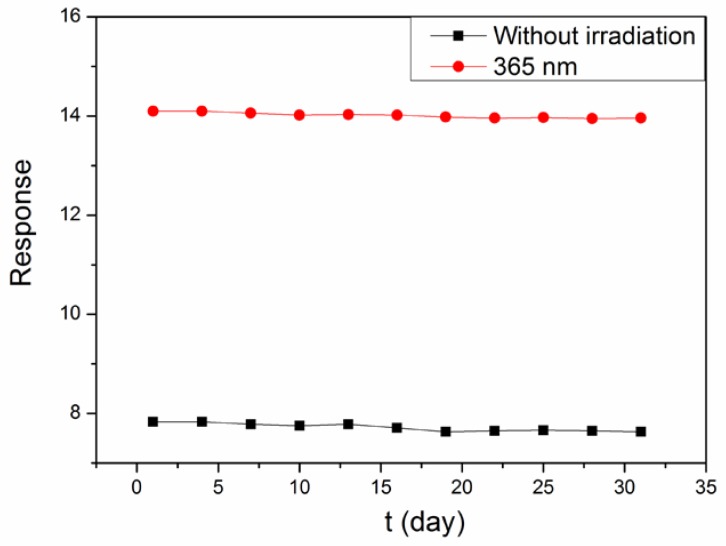
Sensitivity stability of LaFeO_3_ sensor. (black): Without irradiation at 200 °C; (red): 365 nm at 170 °C.

Additionally, the stability of the sensor is also important in practical applications. A good stable sensor will have a larger application scope. To measure the stability of the sensor, the following measurement is performed. The sensor is measured once every 3 days for a month (31 days) and retained in a vacuum bag after every measurement. Figure 10 shows the experiment results of the sensor for stability testing. It is observed that the sensor has a good selectivity whether under irradiation or not. However, the sensor shows better selectivity when used under UV light illumination, and the smaller the wavelength of light, the better selectivity there is.

## 4. Conclusions

LaFeO_3_ is synthesized by the sol-gel method. The sensitivity of the LaFeO_3_ sensor is measured for detecting acetone vapor. The sensor shows a maximum response at 200 °C. As the relative humidity increases, the resistance and sensitivity of sensor are found to decrease. The sensor may be made to work at a low optimum operating temperature by introducing UV light; by doing so, the sensitivity is improved and the optimum operating temperature is decreased. Through the dynamic resistance curve measured under sensor irradiation by UV light, the improved sensitivity mechanism was researched. Additionally, the sensor has a good selectivity toward acetone with an absolute advantage compared with other gases. The sensor can also be used to preliminarily judge if people are diabetic or not, based on the concentration of acetone in their exhaled breath. The sensor shows a better sensitivity and stability when under UV light illumination than that without irradiation.

## Figures and Tables

**Table 1:** Sensing performance toward acetone vapor for other sensors. T_0_ means the optimum operating temperature. C means the concentration of acetone. S means the sensitivity toward acetone vapor.

Author	Materials	T_o_	C	S	Ref.
Epifani et al.	TiO_2_	400	100	1.2	[4]
Bian et al.	TiO_2_	500	10	9	[5]
Bhowmik et al.	TiO_2_	270	10	1.136	[6]
Wang et al.	Au-doped NiO	240	20	7.6	[7]
Wang et al.	W-doped NiO	250	100	198.1	[8]
Wei et al.	ZnO	220	1	7.1	[9]
An et al.	ZnO	220	100	6.0	[10]
Al-Hardan et al.	Cr-doped ZnO	400	500	90	[11]
Peng et al.	ZnO	300	100	18.6	[12]
Rajgure et al.	ZnO	350	2000	92	[13]
Zhang et al.	Co_3_O_4_	150	10	1.7	[14]
Su et al.	Sm-doped α-Fe_2_O_3_	240	0.5	2.3	[15]
Shan et al.	La-doped α-Fe_2_O_3_	240	50	26	[16]
Kim et al.	WO_3_ with both Pd and Au	300	200	152.4	[17]
Chen et al.	WO_3_	300	2	2	[18]
Tomer et al.	WO_3_-SnO_2_	200	50	31.3	[19]
Malik et	Pd-WO_3_	200	25	21.3	[20]
Mishra et al.	SnO_2_	250	10	42	[21]
Jin et al.	SnO_2_	260	25	40	[22]
Punginsang et al.	Co-doped SnO_2_	250	20	36.9	[23]
Singkammo et al.	Ni-Doped SnO_2_	350	200	54.2	[24]
Patil et al.	Co-doped SnO_2_	270	60	32	[25]
Tomer et	Ag-CN	250	50	16.1	[26]
Wu et al.	NdFeO_3_	120	50	300	[27]
Liu et al.	SmFe_0.9_Mg_0.1_O_3_	260	300	353	[28]
Zhang et al.	Ca-doped YbFeO_3_	230	0.1	1.72	[29]
Yang et al.	LaNi_0.5_Ti_0.5_O_3_	350	5	29.3	[30]
Chen et al.	SmFeO_3_	250	380	2.6	[31]
Liu et al.	LaFeO_3_	400	80	204	[32]
Song et al.	LaFeO_3_	240	200	12.2	[33]
Fan et al.	La_0.75_Ba_0.25_FeO_3_	240	50	17	[34]
	LaFeO_3_	200	0.5	1.18	Present work
	LaFeO_3_	200	1	1.22	Present work
	LaFeO_3_	200	2	1.89	Present work
	LaFeO_3_	200	5	3.2	Present work
	LaFeO_3_	200	10	7.83	Present work
	LaFeO_3_ under 365 nm	180	0.5	1.37	Present work
	LaFeO_3_ under 365 nm	180	1	1.85	Present work
	LaFeO_3_ under 365 nm	180	2	3.16	Present work
	LaFeO_3_ under 365 nm	180	5	8.32	Present work
	LaFeO_3_ under 365 nm	180	10	14.1	Present work

**Table 2:** Basic physical status of the four volunteers. FPG means fasting plasma glucose.

Number	Condition	Gender	Age	FPG (mmol/L)
A	Healthy	man	52	4.5
B	Healthy	woman	52	5.3
C	diabetic	man	52	7.8
D	diabetic	woman	52	9.6
